# MULTICOM2 open-source protein structure prediction system powered by deep learning and distance prediction

**DOI:** 10.1038/s41598-021-92395-6

**Published:** 2021-06-23

**Authors:** Tianqi Wu, Jian Liu, Zhiye Guo, Jie Hou, Jianlin Cheng

**Affiliations:** 1 Department of Electrical Engineering and Computer Science, University of Missouri, Columbia, MO 65211 USA; 2 Department of Computer Science, Saint Louis University, St. Louis, MO 63103 USA

**Keywords:** Protein structure predictions, Software

## Abstract

Protein structure prediction is an important problem in bioinformatics and has been studied for decades. However, there are still few open-source comprehensive protein structure prediction packages publicly available in the field. In this paper, we present our latest open-source protein tertiary structure prediction system—MULTICOM2, an integration of template-based modeling (TBM) and template-free modeling (FM) methods. The template-based modeling uses sequence alignment tools with deep multiple sequence alignments to search for structural templates, which are much faster and more accurate than MULTICOM1. The template-free (ab initio or de novo) modeling uses the inter-residue distances predicted by DeepDist to reconstruct tertiary structure models without using any known structure as template. In the blind CASP14 experiment, the average TM-score of the models predicted by our server predictor based on the MULTICOM2 system is 0.720 for 58 TBM (regular) domains and 0.514 for 38 FM and FM/TBM (hard) domains, indicating that MULTICOM2 is capable of predicting good tertiary structures across the board. It can predict the correct fold for 76 CASP14 domains (95% regular domains and 55% hard domains) if only one prediction is made for a domain. The success rate is increased to 3% for both regular and hard domains if five predictions are made per domain. Moreover, the prediction accuracy of the pure template-free structure modeling method on both TBM and FM targets is very close to the combination of template-based and template-free modeling methods. This demonstrates that the distance-based template-free modeling method powered by deep learning can largely replace the traditional template-based modeling method even on TBM targets that TBM methods used to dominate and therefore provides a uniform structure modeling approach to any protein. Finally, on the 38 CASP14 FM and FM/TBM hard domains, MULTICOM2 server predictors (MULTICOM-HYBRID, MULTICOM-DEEP, MULTICOM-DIST) were ranked among the top 20 automated server predictors in the CASP14 experiment. After combining multiple predictors from the same research group as one entry, MULTICOM-HYBRID was ranked no. 5. The source code of MULTICOM2 is freely available at https://github.com/multicom-toolbox/multicom/tree/multicom_v2.0.

## Introduction

Building the high-quality structure of a protein from its sequence is important for studying protein function and has important applications in protein engineering, protein design and drug design. Because the expensive, time-consuming and low-throughput experimental methods for determining protein structures (e.g., X-ray crystallography, nuclear magnetic resonance spectroscopy-NMR, or cryo-electron microscopy) can only be used to solve the structures of a small portion of proteins, fast high-throughput computational protein structure prediction is necessary for constructing structures of the majority of millions of proteins in the nature^1,2^. Various computational methods for protein structure prediction have been proposed, which can be largely classified as template-free modeling (FM, also called ab initio/de novo prediction) and template-based modeling (TBM, also called homology/comparative modeling) methods. When significant structural templates and good template-target sequence alignments are available for a target, template-based modeling methods can generate accurate models for the target. But when there are no good templates, template-free modeling methods are the only viable choice for constructing good structural models without referring to known protein structure templates. Template-free modeling had been studied for decades and progressed very slowly until some breakthroughs in the several few years. The breakthroughs were enabled by two major technical advances, leading to the accurate model prediction for both template-based and template-free targets by template-free modeling methods such as AlphaFold^3^. One advance is the residue-residue co-evolution analysis that provides informative features to improve residue-residue contact/distance predictions for guiding template-free structure reconstruction. Another one is the application of deep learning to protein contact/distance prediction, which dramatically improves the accuracy of contact/distance prediction and therefore substantially enhances the distance-guided template-free modeling^3–6^.

Most recently, the attention mechanism integrated with deep learning was applied to predict residue-residue distances and even 3D coordinates of atoms, which further enhanced the prediction accuracy and interpretability^7,8^. Despite the substantial advances in the field, there are still very few state-of-the-art automated open-source template-based and template-free modeling system available for the community to use^6,9^, hindering the development of new methods and application of protein structure prediction in biomedical research and technology development. Here we introduce our latest open-source protein structure modelling system MULTICOM2 that was recently benchmarked in the 14th Critical Assessment of Techniques for Protein Structure Prediction (CASP14) in 2020. Compared to its previous version (MULTICOM1) tested in CASP13^10^, its template-based modeling method is leaner, more accurate and much faster. Its distance-based template-free modeling method uses our newly developed deep-learning-based inter-residue distance prediction method—DeepDist^11^ and is much more accurate than the contact-based tertiary structure modeling based on CONFOLD2^12^ in MULTICOM1^10^. The server predictors based on MULTICOM2 generated structural models with correct topologies for almost all TBM targets and most FM and FM/TBM targets in the CASP14 experiment and were ranked among the top CASP14 server predictors.

## Results

### Performance of MULTICOM2 in CASP14

The global distance test score (GDT_TS)^13^ and TM-score^14^ are the two standard metrics to evaluate the model quality. The value of TM-score ranges from 0 to 1. A TM-score greater than or equal to 0.5 indicates the predicted model and the native structure have the same fold topology, while a TM-score less than 0.17 means no structural similarity between the predicted model and the native structure. GDT_TS score ranges from 0 to 100% (or simply from 0 to 1), a higher value indicating better model accuracy. Both GDT_TS and TM-score measure the global backbone similarity between a model and the native structure without considering side chain atoms.

Table 1 reports the average TM-score of the top 1 model and the best of top 5 models predicted by MULTICOM-HYBRID, MULTICOM-DEEP, and MULTICOM-DIST for the CASP14 domains, including 58 TBM (regular) domains and 38 FM and FM/TBM (hard) domains. For 58 CASP14 TBM domains, MULTICOM-DEEP ranks highest among three MULTICOM2 sever predictors. It has the average TM-score of 0.730 if it only makes one prediction (one trial) for each domain. Since a model with TM-score > 0.5 is considered to have the correct fold (or topology), we define the success rate as the number of domains for which a correct fold is predicted divided by the total number of domains in consideration. MULTICOM-DEEP predicts correct folds (TM-score > 0.5) for 55 out of 58 domains (i.e., ~ 95% of domains) if it predicts one model domain, and therefore its success rate of one trial is 95% for TBM domains. If the best of the top five models predicted by MULTICOM-DEEP (five trials) is considered, its success rate increases to 98% on the TBM domains. The other two predictors (MULTICOM-HYBRID and MULTICOM-DEEP) has the similar performance.

**Table 1:** TM-scores of models generated by each MULTICOM2 server predictor on CASP14 domains.

Method	Mean TM-score (first model)	Mean TM-score (best of five models)	TM-score > 0.5 (first model)	TM-score > 0.5 (best of top five models)
**(A) On CASP14 58 TBM domains**				
MULTICOM-HYBRID	0.720	0.751	55	57
MULTICOM-DEEP	0.730	0.757	55	57
MULTICOM-DIST	0.702	0.722	53	56
**(B) On CASP14 38 FM and FM/TBM domains**				
MULTICOM-HYBRID	0.514	0.540	21	22
MULTICOM-DEEP	0.512	0.542	20	22
MULTICOM-DIST	0.513	0.554	20	23

The average TM-score of the pure ab initio predictor—MULTICOM-DIST on the TBM domains is 0.702 for one trial, close to that of MULTICOM-HYBRID and MULTICOM-DEEP, indicating that the performance of the distance-based template-free modeling method has reached the performance of the template-based modeling method on the TBM domains. It is remarkable progress considering the substantial gap between the two just a few years ago. Moreover, on the 38 very hard FM and FM/TBM domains, the average TM-score of the three predictors is very close and ranges from 0.512 to 0.514 for one trial. The success rate of MULTICOM-HYBRID on the FM and FM/TBM domains is 55% for one trial. For five trials, its success rate on the FM and FM/TBM domains increases to 58%. MULTICOM-DIST and MULTICOM-DEEP’s performance on the hard domains is similar to MULTICOM-HYBRID, which is not surprising because they are based on the same template-free modeling pipeline of MULTCOM2 for FM and FM/TBM domains.

To fairly compare different predictors that may perform well according to different evaluation metrics, the CASP14 assessors used the summation of Z-scores over the CASP14 targets to rank them^15^, considering three complementary metrics including GDT_TS^13^, Quality Control Score(QCS)^16^ that measures the assembly of secondary structure elements and Molprobity^17^ that evaluates the quality of all the atoms of a model including side chain atoms. The raw score of a model is the weighted average of the three metrics (i.e., GDT_TS + QCS + 0.1 × Molprobity). And it is converted into a Z-score based on the raw scores of all the models for a target. A Z-score less than − 2 is discarded in calculating the summation of Z-scores on all the targets^18^. Since MULTCOM2 is an automated prediction system, we evaluated its performance together with other CASP14 automated server predictors on 38 FM and FM/TBM (hard) domains, excluding CASP14 human predictors involving human intervention in prediction.

**Figure 1:**
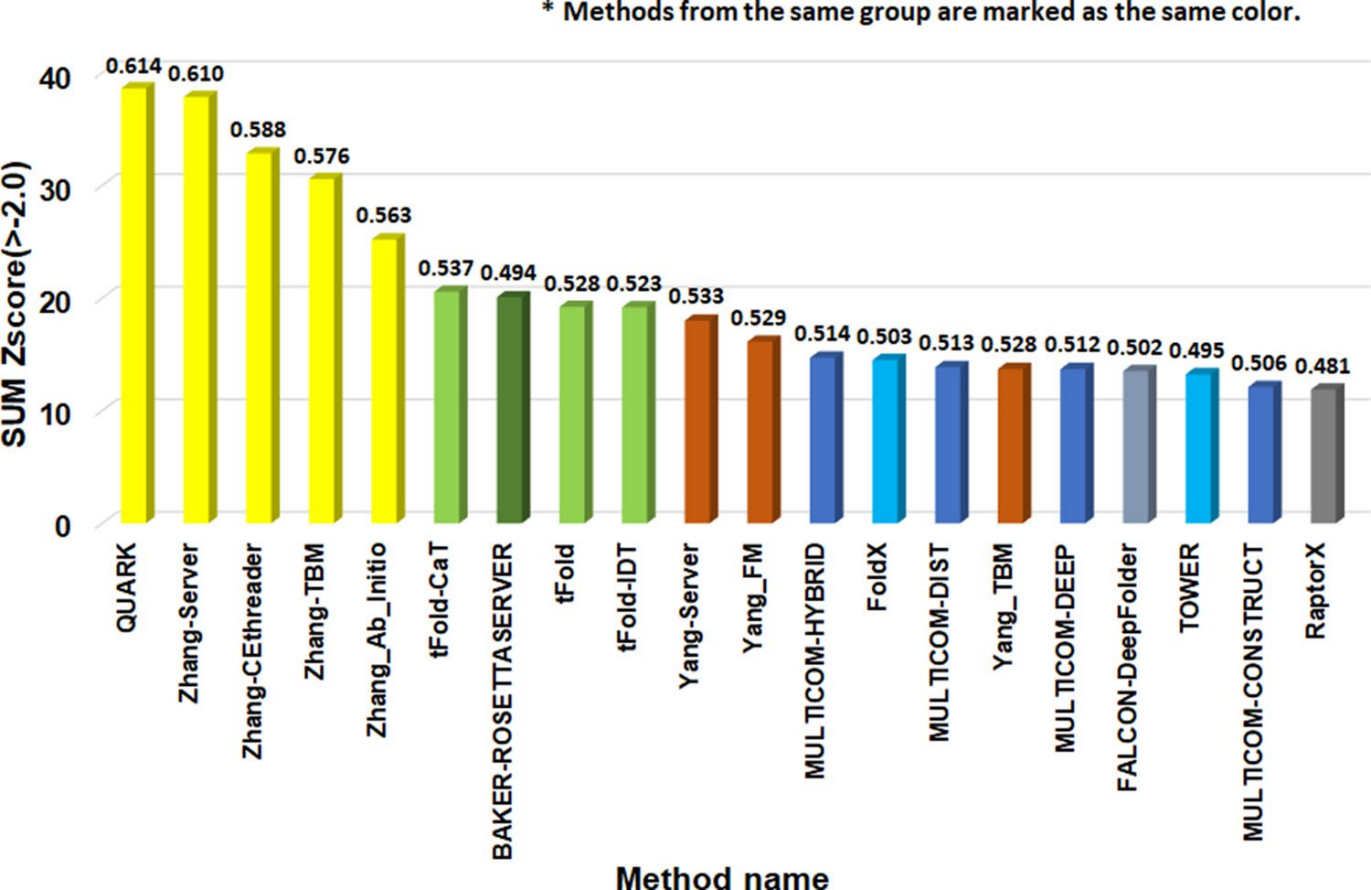
The top 20 server predictors on the 38 CASP14 FM and FM/TBM hard domains ranked by the sum of Z-score calculated according to the CASP14 assessor’s evaluation. The predictors are ranked based on the first models they predicted for 38 CASP14 domains. The predictors from the same group are marked with the same color. The Y-axis denote the sum of the Z-score. The average TM-score of each predictor is reported on top of the bar representing each predictor.

On the 38 CASP14 hard (FM and FM/TBM) domains, all the three MULTICOM2 server predictors, MULTICOM-HYBRID, MULTICOM-DEEP, and MULTICOM-DIST are ranked among the top 20 automated server predictors based on the CASP14’s official sum of Z-scores (Fig. 1). The three MULTICOM2 predictors also performed better than MULTICOM-CONSTRUCT and MULTICOM-CLUSTER—the enhanced version of MULTICOM1^10^ (results not shown). If the multiple predictors from the same research group (e.g., QUARK, ZhangServer, ZhangCEThreader, ZhangTBM, and Zhang_Ab_Initio from Zhang Group) are combined into one entry represented by one predictor with the best performance (e.g., QUARK), MULTICOM-HYBRID representing the MULTICOM2 Group is ranked No. 5. Although the difference in the relatively Z-score of the top five groups is pronounced, the absolute average quality score (e.g., TM-score) of these predictors is much closer. As shown in Fig. 1, the difference of Z-score between MULTICOM-HYBRID and the best predictor of the top four groups—QUARK is 23.9 on the 38 CASP14 FM and FM/TBM hard domains, while the difference of TM-score between them is 0.1.

### Impact of several factors on the performance of the MULTICOM2 system

From the MULTICOM2 results on 58 CASP14 TBM domains in Table 1(A), the pure template-free modeling method of MULTICOM2 (i.e., MULTICOM-DIST) can predict high-quality structures for TBM targets (i.e., average TM-score > 0.7), which is a significant improvement over MULTICOM1. The two integrated MULTICOM2 methods (MULTICOM-HYBRID and MULTICOM-DEEP) that combine the template-free modeling and the traditional template-based homology modeling still outperform MULTICOM-DIST. However, the difference in their performance is not statistically significant.

**Figure 2:**
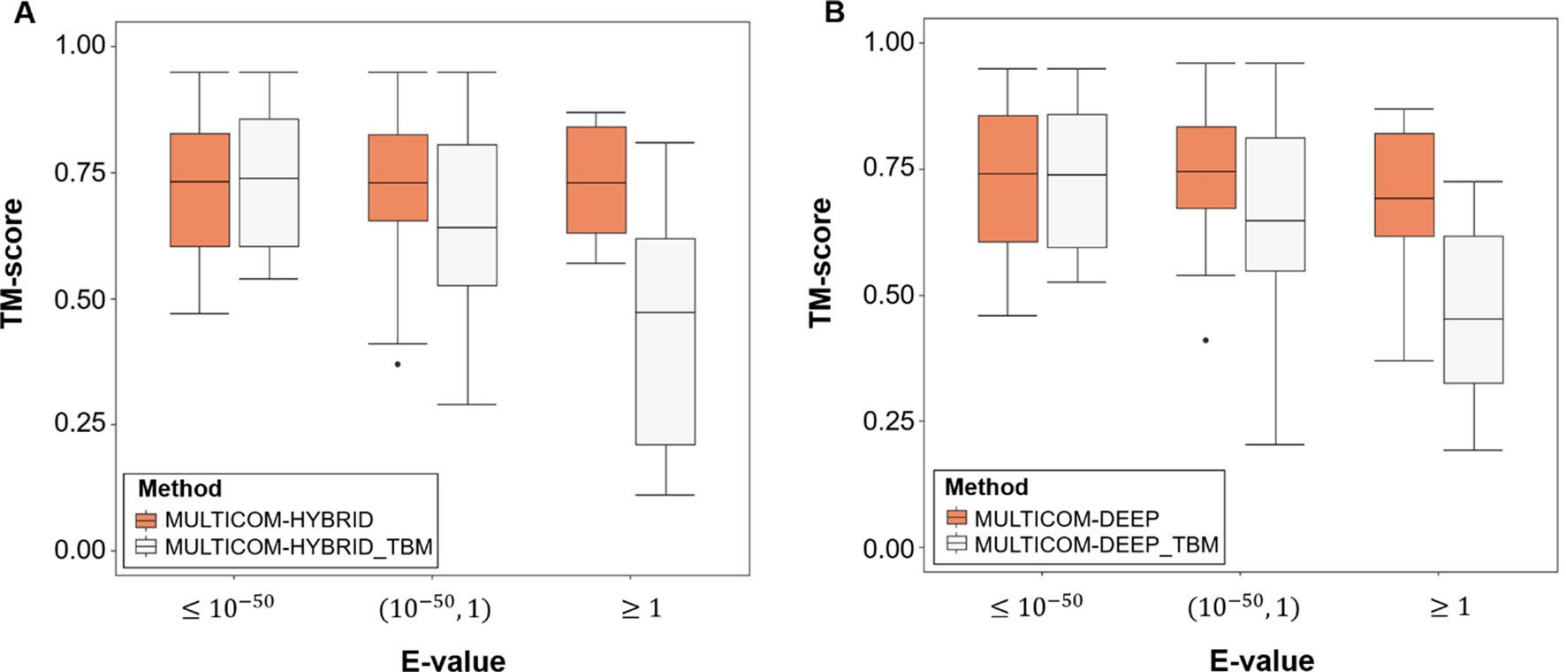
The box plots of the quality of top-1 models from MULTICOM2 integrated server predictors and top-1 models from their templated-based modeling branches on CASP14 TBM domains. (**A**) Comparison between MULTICOM-HYBRID and its templated-based models (MULTICOM-HYBRID_TBM). (**B**) Comparison between MULTICOM-DEEP and its templated-based models (MULTICOM-DEEP_TBM). Top1 templated-based models are selected based on the same model selection methods of MULTICOM-HYBRID and MULTICOM-DEEP mentioned the “Methods” section.

To analyze the impact of the template-based modeling branch of MULTICOM2, we compared the top-1 ranked models from the two integrated methods (MULTICOM-HYBRID and MUTICOM-DEEP) and top-1 ranked template-based models built by their templated-based modeling branch. We found that E-value (i.e., a measure of the significance) of the top template from homology search could be one simple criterion to assess if the templated-based modeling branch can contribute to the final prediction. According to the results on CASP14 TBM domains in Fig. 2 and Table 2, when the top template hits have E-value ≤ 10^−50^ (i.e., highly significant), the top templated-based models from the template-based modeling branch have the quality comparable to that of the final top models predicted by the integrated system. For MULTICOM-HYRBID, the average TM-score of top 1 models from the template-based branch and the integrated system are 0.738 and 0.731, respectively. For MULTICOM-DEEP, the average TM-score of top 1 models from the template-based branch and the final integrated system are 0.739 and 0.740, respectively. In this situation, the template-based models sometimes are selected as top-1 models of the integrated system. When the top template hits have E-value between 10^−50^ and 1, the average TM-score of top templated-based models from the template-based modeling branch is 0.642 for MULTICOM-HYBRID_TBM and 0.648 for MULTICOM-DEEP_TBM, which is much lower than the scores (0.730 and 0.746) of top models of the integrated systems (MULTICOM-HYBRID and MULTICOM-DEEP), indicating that the template-based modeling has a much less impact in this situation. When the top template hits have E-value ≥ 1 (i.e., insignificant), the average TM-score of the top-1 models from MULTICOM-HYBRID and MULTICOM-DEEP is 0.730 and 0.692, much higher than 0.473 and 0.452 of the top-1 template models from the template-based modeling branch, indicating that the template-based models are not useful in this situation. The detailed per-domain comparison is shown in the Supplementary Table S1. Overall, the performance of template-based modeling method on TBM domains quickly decreases as templates become less significant, while the integrated prediction system such as MULTICOM-HYBRID still maintains a high prediction accuracy, indicating that the template-free modeling method can achieve a rather good accuracy on TBM domains across the board regardless of their difficulty. In fact, according to the analysis on 74 full-length CASP14 targets, MULTICOM-HYBRID selected template-free modeling models as top-1 models for 55 targets including ones having highly significant templates (see Table S2 for details).

**Table 2:** Comparison of the quality of top-1 models from MULTICOM2 integrated server predictors (MULTICOM-HYBRID and MULTICOM-DEEP) and top-1 models from their templated-based modeling branches (MULTICOM-HYBRID_TBM and MULTICOM-DEEP_TBM) on 54 CASP14 TBM domains with native structures available for this analysis. The central line in the box marks the average TM-score values of top 1 models.

Method	MULTICOM-HYBRID	MULTICOM-HYBRID_TBM	MULTICOM-DEEP	MULTICOM-DEEP_TBM
E-value $\le$ 10^–50^	0.731	0.738	0.740	0.739
10^–50^ $<$ E-value $<$ 1	0.730	0.642	0.746	0.648
E-value $\ge$ 1	0.730	0.473	0.692	0.452

Top-1 templated-based models are selected based on the same model selection method of MULTICOM-HYBRID and MULTICOM-DEEP described in the “Methods” section.

**Figure 3:**
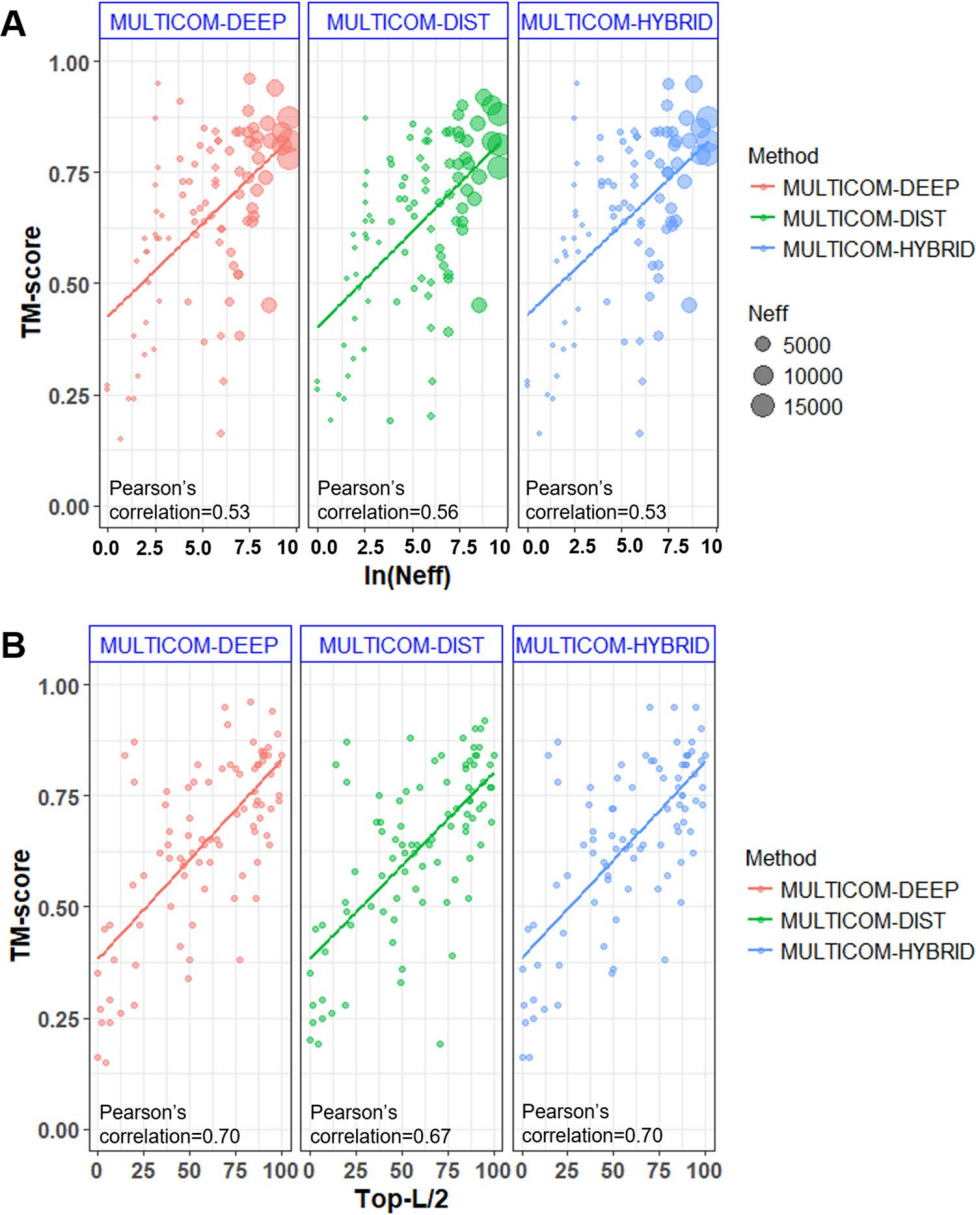
Impact of Neff and the accuracy of the inter-residue distance prediction on the model quality of the MUTLICOM2 system on 91 CASP14 domains whose experimental structures are available for analysis. (**A**) Logarithm of Neff of MSA vs. the quality of models built from three MULTICOM server predictors (MULTICOM-DEEP, MULTICOM-DIST and MULTICOM-HYBRID). The size of a dot is proportional to the value of Neff. (**B**) The precision of top L/2 long-range contact predictions vs. the quality of models.

While the significance of templates determines the quality of template-based models and their contribution to the whole system, the number of effective sequence (Neff)^19^ or more precisely quality of multiple sequence alignments (MSA) and the accuracy of the inter-residue distance prediction are critical for the template-free modeling branch and the entire MULTICOM2 prediction system. Fig. 3A shows the comparison between the quality of MSA roughly measured by the logarithm of Neff and the quality (i.e., TM-score) of the top-1 models built by the three MULTICOM2 server predictors. There is a moderate correlation between the two on all 91 CASP14 domains whose native structures are available. The Pearson’s correlation coefficients of MULTICOM-DIST, MULTICOM-HYBRID and MULTICOM-DEEP are 0.56, 0.53 and 0.53, respectively. Figure 3B shows a stronger correlation between the distance prediction accuracy (measured as the precision of top L/2 long-range contact predictions^20^; L: sequence length) and the quality of the top-1 models. Pearson’s correlation coefficient for MULTICOM-DIST, MULTICOM-HYBRID, and MULTICOM-DEEP is 0.67, 0.70, and 0.70, respectively. The detailed results for each CASP domain are shown in the Supplementary Table S3.

### Runtime of MULTICOM2

It typically requires 12 hours for a protein of typical length (e.g., 300 residues) for MULTICOM2 to complete the entire modeling process on an Intel(R) Xeon(R) CPU E5-2660 v3 10-core processor, which is about two times faster than MULTICOM1. With hundreds of cores available on a computer cluster, MULTICOM2 can be used to predict structures of many proteins per day and therefore is applicable to the genome-level protein structure modeling.

## Conclusion

We develop and release our latest automated protein structure prediction system (MULTICOM2) as an open-source software package for the community to use. MULTCOM2 is leaner, more accurate, and much faster than MULTICOM1. It was ranked among top automated server predictors in the latest 2020 CASP14 experiment. It can predict correct folds for almost all regular targets and a majority of very hard targets. Its template-free modeling without using any known structures as templates can work well on both regular template-based modeling targets and hard template-free modeling targets, which provides a uniform, reliable approach to modeling the structure of any protein. Therefore, it can be a useful tool for both the current development and application of protein structure prediction methods. While the inter-residue distance prediction can be converted to tertiary structures, significant modeling effort is still needed to do the conversion. The recent success of AlphaFold2^8^ in CASP14 demonstrates that an end-to-end deep learning system with the attention mechanism can directly predict the tertiary structure of a protein from its multiple sequence alignment, skipping the distance-to-structure conversion. In the future, we plan to develop deep learning-based end-to-end protein structure prediction methods and add them into the next release of MULTICOM.

## Methods

**Figure 4:**
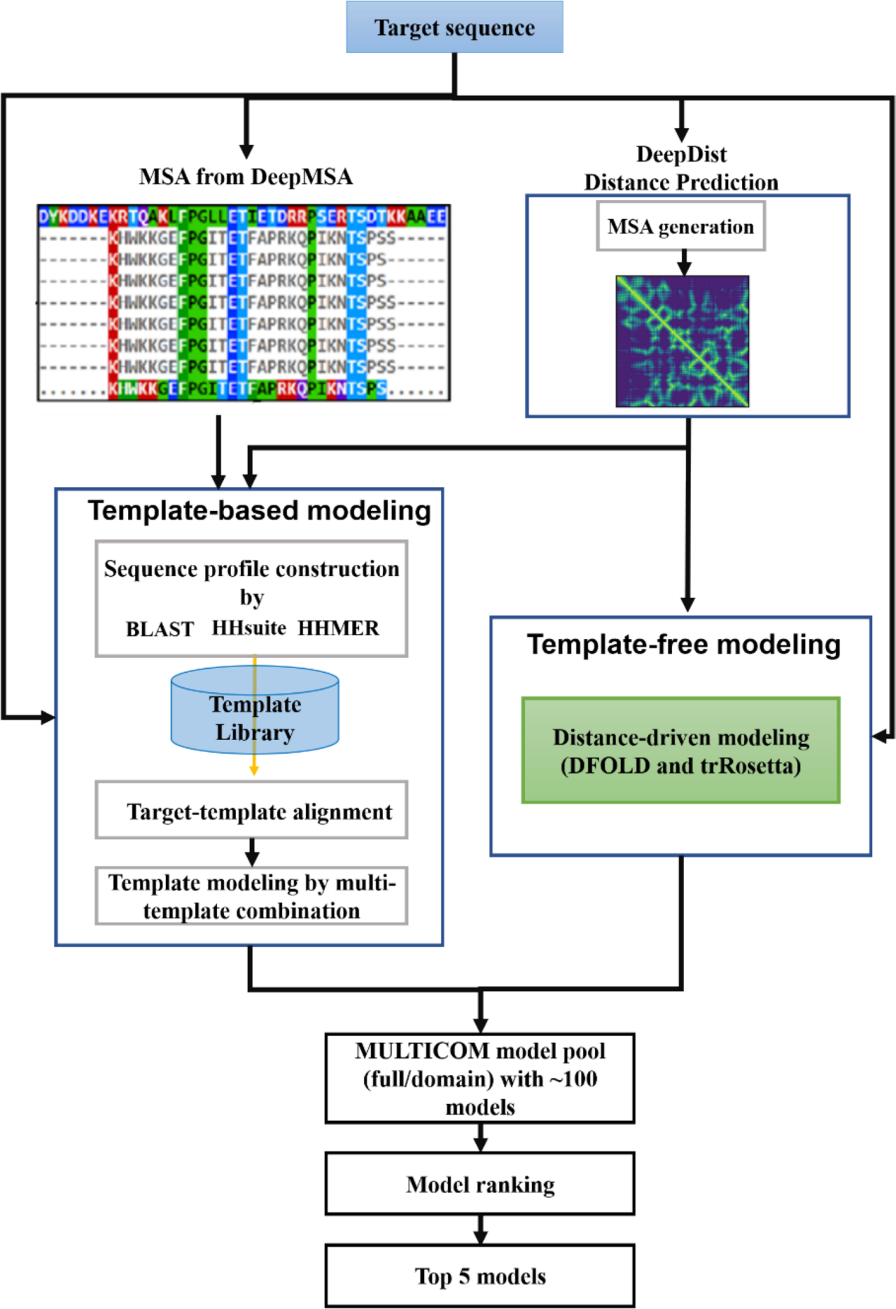
Flowchart of the MULTICOM2 system consisting of template-based, template-free modeling methods and model ranking (quality assessment).

Figure 4 illustrates the flowchart of the MULTICOM2 system. It is an automated and integrated protein modeling system that combines template-based modeling, template-free modeling, and model quality assessment.

Given a target sequence, MULTICOM2 searches it against the non-redundant protein sequence databases to build target sequence profiles such as position-specific scoring matrices (PSSM) and hidden Markov models (HMMs) using HH-suite^21^, PSI-BLAST^1^, and HMMER^22^. Each profile is searched against a template library in order to identify a list of structurally similar templates and their sequence alignments with the target sequence. The pairwise target-template alignments are combined into multi-template alignments between the target and the multiple templates if the structures of the templates are consistent^23,24^. Multi-template alignments along with the template structures are fed into Modeller^25^ to build the structural models for the target protein. Different from MULTICOM1^10^ that uses more than a dozen sequence alignment tools and relatively slow threading tools (e.g. COMPASS^26^, FFAS^27^, SAM^28^, PRC^29^, HH-suite^21^, PSI-BLAST^1^, HMMER^22^, RaptorX^30^, I-TASSER/MUSTER^9,31^), MULTICOM2 only uses HH-suite^21^, PSI-BLAST^1^, and HMMER^22^ to build profiles, identify protein templates and generate target-template sequence alignments. Among those tools, hhblits and hhsearch in HH-suite are much more sensitive than PSI-BLAST and HMMER and play an essential role in the template identification. Therefore, the template search process in MULTICOM2 is much faster than MULTICOM1. In order to further improve the sensitivity of template identification, MULTICOM2 applies DeepMSA^32^ to search the target sequence against large sequence databases (Uniclust30^33^, Uniref90, metagenomics sequence database^34^) for generating the deep multiple sequence alignments, which are used to build profiles to identify templates for template-based modeling.

In parallel to the template-based modeling, the target sequence is also used as input for a deep learning distance predictor—DeepDist^35^ to predict inter-residue distances. The predicted distance maps are used by DFOLD^35,36^ (our in-house distance-guided ab initio modeling tool based on Crystallography and NMR System—CNS^37^) and trRosetta^6^ to generate template-free models. DFOLD differs from CONFOLD2 (also based on CNS) that takes binary contacts as input to build tertiary structures. To use trRosetta, the distance maps predicted by DeepDist are used to substitute the default distance maps generated by trRosetta from the multiple sequence alignments before ab initio tertiary structure modeling. The DeepDist’s distance prediction is also used to select templates based on their matching with the predicted distance maps for template-based modeling. By default, about 100 template-free and template-based models are constructed for a target. The models are ranked by three different model quality assessment methods: APOLLO^38^ of ranking models based on their pairwise structural similarity, SBROD^39^—a single model energy function of ranking models, and the distance-based ranking based on the similarity between the predicted distance maps and the distance maps of a model^40^. Any one of the three rankings or their consensus can be used to select top models for the target.

Based on the target-template sequence alignment generated by HH-suite, MULTICOM2 also predicts if a target needs to be split into multiple domains or modeling units. If some region (> 40 residues) of a target does not have significant templates but other regions have, it will be split into multiple template-based and template-free domains according to the alignment. The structural models for each domain are then predicted by the same pipeline above. The top five ranked models from each domain are joined into full-length models for the multi-domain target by Modeller^25^ or AIDA^41^.

In order to evaluate different modeling options of MULTICOM2, three predictors based on MULTICOM2—MULTCOM-HYBRID, MULTICOM-DEEP, and MULTICOM-DIST participated in the CASP14 experiment. MULTICOM-HYBRID and MULTICOM-DEEP used both the template-based and template-free modeling and differed only in model ranking. MULTICOM-HYBRID primarily used APOLLO^38^ to rank models, whereas MULTICOM-DEEP primarily used the average ranking of SBROD^39^ and the distance-based ranking (the matching between a model and the predicted distance map) to select final models. MULTICOM-DIST skipped template-based tertiary structure modeling entirely and only used the template-free modeling to generate tertiary structure models and used SBROD^39^ to rank them, even though it still used template-target alignments to identify domain boundaries if needed.

## Supplementary Information

Supplementary Information.
