# Supplementary material for: MULTICOM2 open-source protein structure prediction system powered by deep learning and distance prediction
Source: Sci Rep. 2021 Jun 23;11:13155. doi: 10.1038/s41598-021-92395-6 (PMC8222248; doi:10.1038/s41598-021-92395-6)
Supplement: Supplementary file 1 — Supplementary Information. [file 41598_2021_92395_MOESM1_ESM.docx]

**Table S1.** Per-domain comparison of the quality of top-1 template-based models with the quality of the top-1 models from two MULTICOM2 server predictors on 54 CASP14 TBM domains with native structures available. E-value of the top template hit is also listed. The model quality is calculated as TM-score. (A) Results for MULTICOM-HYBRID. (A) Results for MULTICOM-DEEP.

**(A) MULTICOM-HYBRID**

| Domain | E-value of Top1 Hit | MULTICOM-HYBRID | MULTICOM-HYBRID _TBM |
| --- | --- | --- | --- |
| T1036s1-D1 | 2.6E-227 | 0.83 | 0.89 |
| T1050-D1 | 5.4E-61 | 0.79 | 0.78 |
| T1050-D2 | 5.4E-61 | 0.82 | 0.82 |
| T1050-D3 | 5.4E-61 | 0.87 | 0.87 |
| T1052-D1 | 3.10E-51 | 0.95 | 0.95 |
| T1068-D1 | 3.6E-68 | 0.57 | 0.54 |
| T1076-D1 | 3.7E-66 | 0.95 | 0.94 |
| T1079-D1 | 5.5E-96 | 0.75 | 0.75 |
| T1092-D1 | 2.8E-80 | 0.47 | 0.59 |
| T1092-D2 | 2.8E-80 | 0.79 | 0.79 |
| T1093-D2 | 3.7E-95 | 0.64 | 0.64 |
| T1094-D1 | 3.2E-53 | 0.59 | 0.59 |
| T1095-D1 | 2.5E-97 | 0.67 | 0.64 |
| T1099-D1 | 7.3E-65 | 0.55 | 0.54 |
| T1024-D1 | 2.3E-34 | 0.85 | 0.84 |
| T1024-D2 | 2.3E-34 | 0.79 | 0.81 |
| T1025-D1 | 7.3E-24 | 0.9 | 0.89 |
| T1028-D1 | 1.3E-27 | 0.82 | 0.82 |
| T1030-D1 | 1.1E-18 | 0.64 | 0.36 |
| T1030-D2 | 1.1E-18 | 0.37 | 0.29 |
| T1032-D1 | 4.8E-13 | 0.63 | 0.56 |
| T1045s1-D1 | 5.3E-33 | 0.95 | 0.95 |
| T1045s2-D1 | 5.7E-15 | 0.8 | 0.67 |
| T1052-D2 | 7.90E-15 | 0.77 | 0.64 |
| T1054-D1 | 1.6E-15 | 0.62 | 0.41 |
| T1056-D1 | 5.4E-23 | 0.57 | 0.57 |
| T1057-D1 | 4.8E-47 | 0.84 | 0.83 |
| T1060s2-D1 | 0.00000095 | 0.67 | 0.72 |
| T1060s3-D1 | 0.061 | 0.73 | 0.57 |
| T1061-D3 | 7.90E-15 | 0.68 | 0.61 |
| T1067-D1 | 4.2E-25 | 0.54 | 0.48 |
| T1073-D1 | 0.0057 | 0.77 | 0.72 |
| T1078-D1 | 0.000085 | 0.84 | 0.51 |
| T1083-D1 | 0.052 | 0.83 | 0.51 |
| T1084-D1 | 0.025 | 0.82 | 0.75 |
| T1087-D1 | 0.65 | 0.41 | 0.67 |
| T1089-D1 | 1.1E-31 | 0.81 | 0.81 |
| T1091-D1 | 2.70E-22 | 0.74 | 0.56 |
| T1091-D2 | 1.10E-07 | 0.77 | 0.55 |
| T1091-D3 | 1.10E-07 | 0.72 | 0.5 |
| T1091-D4 | 1.10E-07 | 0.84 | 0.8 |
| T1100-D1 | 8.8E-20 | 0.67 | 0.44 |
| T1100-D2 | 8.8E-20 | 0.64 | 0.54 |
| T1101-D1 | 1.3E-23 | 0.84 | 0.7 |
| T1101-D2 | 1.3E-23 | 0.75 | 0.82 |
| T1026-D1 | 30 | 0.57 | 0.76 |
| T1034-D1 | 18 | 0.87 | 0.81 |
| T1046s2-D1 | 230 | 0.63 | 0.62 |
| T1047s2-D2 | 20 | 0.84 | 0.11 |
| T1058-D2 | 1.3 | 0.64 | 0.49 |
| T1065s1-D1 | 550 | 0.83 | 0.43 |
| T1070-D2 | 41 | 0.87 | 0.62 |
| T1070-D3 | 41 | 0.6 | 0.21 |
| T1070-D4 | 41 | 0.72 | 0.21 |

**(B) MULTICOM-DEEP**

| Domain | E-value of Top1 Hit | MULTICOM-HYBRID | MULTICOM-HYBRID _TBM |
| --- | --- | --- | --- |
| T1036s1-D1 | 5.70E-226 | 0.91 | 0.91 |
| T1050-D1 | 5.40E-61 | 0.78 | 0.78 |
| T1050-D2 | 5.40E-61 | 0.82 | 0.82 |
| T1050-D3 | 5.40E-61 | 0.87 | 0.87 |
| T1052-D1 | 3.10E-51 | 0.95 | 0.95 |
| T1068-D1 | 1.00E-67 | 0.6 | 0.53 |
| T1076-D1 | 3.70E-66 | 0.94 | 0.94 |
| T1079-D1 | 5.50E-96 | 0.82 | 0.75 |
| T1092-D1 | 6.10E-80 | 0.46 | 0.59 |
| T1092-D2 | 6.10E-80 | 0.8 | 0.80 |
| T1093-D2 | 3.70E-95 | 0.62 | 0.64 |
| T1095-D1 | 2.70E-104 | 0.65 | 0.59 |
| T1099-D1 | 2.10E-77 | 0.55 | 0.59 |
| T1094-D1 | E-147 | 0.59 | 0.58 |
| T1024-D1 | 2.30E-34 | 0.84 | 0.84 |
| T1024-D2 | 2.30E-34 | 0.81 | 0.81 |
| T1025-D1 | 7.30E-24 | 0.89 | 0.89 |
| T1028-D1 | 2.70E-34 | 0.82 | 0.82 |
| T1032-D1 | 2.00E-20 | 0.65 | 0.57 |
| T1034-D1 | 8.40E-06 | 0.86 | 0.85 |
| T1045s1-D1 | 5.40E-31 | 0.96 | 0.96 |
| T1045s2-D1 | 0.00071 | 0.8 | 0.63 |
| T1047s2-D2 | 0.016 | 0.84 | 0.20 |
| T1052-D2 | 1.70E-16 | 0.76 | 0.63 |
| T1054-D1 | 2.80E-17 | 0.64 | 0.43 |
| T1056-D1 | 5.30E-16 | 0.57 | 0.56 |
| T1057-D1 | 3.80E-48 | 0.85 | 0.85 |
| T1058-D2 | 0.34 | 0.71 | 0.57 |
| T1060s2-D1 | 9.50E-07 | 0.66 | 0.72 |
| T1060s3-D1 | 0.061 | 0.74 | 0.57 |
| T1061-D3 | 2.70E-10 | 0.68 | 0.61 |
| T1067-D1 | 2.00E-16 | 0.54 | 0.48 |
| T1073-D1 | 1.70E-20 | 0.78 | 0.56 |
| T1078-D1 | 8.50E-05 | 0.81 | 0.51 |
| T1087-D1 | 0.65 | 0.41 | 0.67 |
| T1089-D1 | 1.40E-26 | 0.81 | 0.81 |
| T1091-D1 | 3.90E-37 | 0.72 | 0.55 |
| T1091-D2 | 1.10E-07 | 0.76 | 0.55 |
| T1091-D3 | 1.10E-07 | 0.73 | 0.50 |
| T1091-D4 | 1.10E-07 | 0.84 | 0.80 |
| T1100-D1 | 8.80E-20 | 0.67 | 0.44 |
| T1100-D2 | 8.80E-20 | 0.64 | 0.54 |
| T1101-D1 | 1.30E-23 | 0.84 | 0.70 |
| T1101-D2 | 1.30E-23 | 0.74 | 0.82 |
| T1026-D1 | 5.1 | 0.57 | 0.67 |
| T1030-D1 | 4.8 | 0.64 | 0.34 |
| T1030-D2 | 4.8 | 0.37 | 0.32 |
| T1046s2-D1 | 2.30E+02 | 0.65 | 0.62 |
| T1065s1-D1 | 5.50E+02 | 0.82 | 0.43 |
| T1070-D2 | 30 | 0.87 | 0.60 |
| T1070-D3 | 30 | 0.61 | 0.22 |
| T1070-D4 | 30 | 0.72 | 0.19 |
| T1083-D1 | 2.20E+02 | 0.82 | 0.40 |
| T1084-D1 | 88 | 0.85 | 0.73 |

**Table S2.** The modeling type (FM: template-free modeling or TBM: template-based modeling) of the top-1 models selected by MULTICOM-HYBRID for the 74 CASP14 targets. The e-value of the top-1 template hit for each target is also reported. MULTICOM-HYBRID used template-free modeling to generate the first model for 55 out of 74 targets including some that have significant templates (e-value < E-10).

| Target | E-value of Top 1 Template Hit | Type of Top 1 Model | Target | E-value of Top 1 Template Hit | Type of Top1 Model |
| --- | --- | --- | --- | --- | --- |
| T1068 | 3.60E-68 | FM | T1072s1 | 2.90E+01 | FM |
| T1076 | 3.70E-66 | FM | T1026 | 3.00E+01 | FM |
| T1085 | 2.30E-37 | FM | T1088 | 3.60E+01 | FM |
| T1098 | 7.40E-34 | FM | T1070 | 4.10E+01 | FM |
| T1063 | 2.00E-32 | FM | T1027 | 5.00E+01 | FM |
| T1067 | 4.20E-25 | FM | T1033 | 1.00E+02 | FM |
| T1101 | 1.30E-23 | FM | T1029 | 1.30E+02 | FM |
| T1100 | 8.80E-20 | FM | T1043 | 1.30E+02 | FM |
| T1030 | 1.10E-18 | FM | T1096 | 1.50E+02 | FM |
| T1054 | 1.60E-15 | FM | T1037 | 1.60E+02 | FM |
| T1045s2 | 5.70E-15 | FM | T1090 | 1.70E+02 | FM |
| T1032 | 4.80E-13 | FM | T1065s2 | 1.90E+02 | FM |
| T1075 | 1.40E-12 | FM | T1046s2 | 2.30E+02 | FM |
| T1051 | 1.80E-12 | FM | T1074 | 2.30E+02 | FM |
| T1077 | 4.60E-09 | FM | T1042 | 3.20E+02 | FM |
| T1060s2 | 9.50E-07 | FM | T1065s1 | 5.50E+02 | FM |
| T1078 | 8.50E-05 | FM | T1049 | 5.90E+02 | FM |
| T1066s2 | 3.00E-03 | FM | T1046s1 | 8.90E+02 | FM |
| T1073 | 5.70E-03 | FM | T1036s1 | 2.60E-227 | TBM |
| T1084 | 2.50E-02 | FM | T1095 | 2.50E-97 | TBM |
| T1048 | 3.30E-02 | FM | T1079 | 5.50E-96 | TBM |
| T1083 | 5.20E-02 | FM | T1093 | 3.70E-95 | TBM |
| T1060s3 | 6.10E-02 | FM | T1092 | 2.80E-80 | TBM |
| T1087 | 6.50E-01 | FM | T1099 | 7.30E-65 | TBM |
| T1041 | 9.60E-01 | FM | T1050 | 5.40E-61 | TBM |
| T1058 | 1.30E+00 | FM | T1069s1 | 3.50E-59 | TBM |
| T1069s2 | 2.20E+00 | FM | T1094 | 3.20E-53 | TBM |
| T1072s2 | 2.50E+00 | FM | T1057 | 4.80E-47 | TBM |
| T1055 | 2.70E+00 | FM | T1066s1 | 1.50E-43 | TBM |
| T1040 | 2.80E+00 | FM | T1071 | 1.60E-40 | TBM |
| T1047s1 | 2.90E+00 | FM | T1024 | 2.30E-34 | TBM |
| T1064 | 3.10E+00 | FM | T1045s1 | 5.30E-33 | TBM |
| T1035 | 4.40E+00 | FM | T1086 | 5.60E-32 | TBM |
| T1082 | 6.70E+00 | FM | T1089 | 1.10E-31 | TBM |
| T1053 | 1.50E+01 | FM | T1028 | 1.30E-27 | TBM |
| T1034 | 1.80E+01 | FM | T1025 | 7.30E-24 | TBM |
| T1047s2 | 2.00E+01 | FM | T1056 | 5.40E-23 | TBM |

**Table S3.** Precision of Top L/2 long-range contact predictions VS the quality of top-1 models from three MULTICOM2 server predictors on 91 CASP14 domains with native structures available (L: sequence length). (A) MUTLICOM-DIST. (B) MULTICOM-HYBRID. (C)MULTICOM-DEEP.

**(A) MULTICOM-DIST (B) MULTICOM-HYBRID (C)MULTICOM-DEEP**

| DOMAIN | Top-L/2(%) | TM-score |  | DOMAIN | Top-L/2 (%) | TM-score |  | DOMAIN | Top-L/2 (%) | TM-score |
| --- | --- | --- | --- | --- | --- | --- | --- | --- | --- | --- |
| T1024-D1 | 92.78 | 0.9 |  | T1024-D1 | 92.78 | 0.85 |  | T1024-D1 | 92.78 | 0.84 |
| T1024-D2 | 84.85 | 0.82 |  | T1024-D2 | 84.85 | 0.79 |  | T1024-D2 | 84.85 | 0.81 |
| T1025-D1 | 97.67 | 0.77 |  | T1025-D1 | 97.67 | 0.9 |  | T1025-D1 | 97.67 | 0.89 |
| T1026-D1 | 24.66 | 0.58 |  | T1026-D1 | 24.66 | 0.57 |  | T1026-D1 | 24.66 | 0.57 |
| T1027-D1 | 50 | 0.36 |  | T1027-D1 | 50 | 0.36 |  | T1027-D1 | 50 | 0.38 |
| T1028-D1 | 86.99 | 0.74 |  | T1028-D1 | 86.99 | 0.82 |  | T1028-D1 | 86.99 | 0.82 |
| T1029-D1 | 6.35 | 0.46 |  | T1029-D1 | 6.35 | 0.46 |  | T1029-D1 | 6.35 | 0.46 |
| T1030-D1 | 66.23 | 0.65 |  | T1030-D1 | 66.23 | 0.64 |  | T1030-D1 | 66.23 | 0.64 |
| T1030-D2 | 20 | 0.49 |  | T1030-D2 | 20 | 0.37 |  | T1030-D2 | 20 | 0.37 |
| T1031-D1 | 6.25 | 0.29 |  | T1031-D1 | 6.25 | 0.29 |  | T1031-D1 | 6.25 | 0.29 |
| T1032-D1 | 56.47 | 0.62 |  | T1032-D1 | 56.47 | 0.63 |  | T1032-D1 | 56.47 | 0.65 |
| T1033-D1 | 12 | 0.26 |  | T1033-D1 | 12 | 0.27 |  | T1033-D1 | 12 | 0.26 |
| T1034-D1 | 92.31 | 0.86 |  | T1034-D1 | 92.31 | 0.87 |  | T1034-D1 | 92.31 | 0.86 |
| T1035-D1 | 19.61 | 0.78 |  | T1035-D1 | 19.61 | 0.78 |  | T1035-D1 | 19.61 | 0.78 |
| T1036s1-D1 | 70.55 | 0.19 |  | T1036s1-D1 | 70.55 | 0.83 |  | T1036s1-D1 | 70.55 | 0.91 |
| T1037-D1 | 36.14 | 0.69 |  | T1037-D1 | 36.14 | 0.73 |  | T1037-D1 | 36.14 | 0.73 |
| T1038-D1 | 49.12 | 0.33 |  | T1038-D1 | 49.12 | 0.35 |  | T1038-D1 | 49.12 | 0.34 |
| T1038-D2 | 86.84 | 0.61 |  | T1038-D2 | 86.84 | 0.6 |  | T1038-D2 | 86.84 | 0.6 |
| T1039-D1 | 1.23 | 0.28 |  | T1039-D1 | 1.23 | 0.28 |  | T1039-D1 | 1.23 | 0.27 |
| T1040-D1 | 1.54 | 0.24 |  | T1040-D1 | 1.54 | 0.24 |  | T1040-D1 | 1.54 | 0.24 |
| T1041-D1 | 51.24 | 0.62 |  | T1041-D1 | 51.24 | 0.63 |  | T1041-D1 | 51.24 | 0.62 |
| T1042-D1 | 22.46 | 0.46 |  | T1042-D1 | 22.46 | 0.44 |  | T1042-D1 | 22.46 | 0.46 |
| T1043-D1 | 4.05 | 0.19 |  | T1043-D1 | 4.05 | 0.16 |  | T1043-D1 | 4.05 | 0.15 |
| T1045s1-D1 | 83.12 | 0.88 |  | T1045s1-D1 | 83.12 | 0.95 |  | T1045s1-D1 | 83.12 | 0.96 |
| T1045s2-D1 | 93.98 | 0.78 |  | T1045s2-D1 | 93.98 | 0.8 |  | T1045s2-D1 | 93.98 | 0.8 |
| T1046s1-D1 | 61.11 | 0.59 |  | T1046s1-D1 | 61.11 | 0.61 |  | T1046s1-D1 | 61.11 | 0.6 |
| T1046s2-D1 | 64.79 | 0.64 |  | T1046s2-D1 | 64.79 | 0.63 |  | T1046s2-D1 | 64.79 | 0.65 |
| T1047s1-D1 | 77.36 | 0.39 |  | T1047s1-D1 | 77.36 | 0.38 |  | T1047s1-D1 | 77.36 | 0.38 |
| T1047s2-D1 | 98.55 | 0.77 |  | T1047s2-D1 | 98.55 | 0.77 |  | T1047s2-D1 | 98.55 | 0.75 |
| T1047s2-D2 | 100 | 0.84 |  | T1047s2-D2 | 100 | 0.84 |  | T1047s2-D2 | 100 | 0.84 |
| T1047s2-D3 | 88.1 | 0.7 |  | T1047s2-D3 | 88.1 | 0.69 |  | T1047s2-D3 | 88.1 | 0.7 |
| T1049-D1 | 85.07 | 0.67 |  | T1049-D1 | 85.07 | 0.67 |  | T1049-D1 | 85.07 | 0.67 |
| T1050-D1 | 52.17 | 0.76 |  | T1050-D1 | 52.17 | 0.79 |  | T1050-D1 | 52.17 | 0.78 |
| T1050-D2 | 54.43 | 0.88 |  | T1050-D2 | 54.43 | 0.82 |  | T1050-D2 | 54.43 | 0.82 |
| T1050-D3 | 84.38 | 0.81 |  | T1050-D3 | 84.38 | 0.87 |  | T1050-D3 | 84.38 | 0.87 |
| T1052-D1 | 69.26 | 0.6 |  | T1052-D1 | 69.26 | 0.95 |  | T1052-D1 | 69.26 | 0.95 |
| T1052-D2 | 37.38 | 0.75 |  | T1052-D2 | 37.38 | 0.77 |  | T1052-D2 | 37.38 | 0.76 |
| T1052-D3 | 55 | 0.64 |  | T1052-D3 | 55 | 0.65 |  | T1052-D3 | 55 | 0.6 |
| T1053-D1 | 50 | 0.64 |  | T1053-D1 | 50 | 0.72 |  | T1053-D1 | 50 | 0.7 |
| T1053-D2 | 86.05 | 0.77 |  | T1053-D2 | 86.05 | 0.73 |  | T1053-D2 | 86.05 | 0.73 |
| T1054-D1 | 93.06 | 0.64 |  | T1054-D1 | 93.06 | 0.62 |  | T1054-D1 | 93.06 | 0.64 |
| T1055-D1 | 49.18 | 0.68 |  | T1055-D1 | 49.18 | 0.66 |  | T1055-D1 | 49.18 | 0.66 |
| T1056-D1 | 78.82 | 0.56 |  | T1056-D1 | 78.82 | 0.57 |  | T1056-D1 | 78.82 | 0.57 |
| T1057-D1 | 90.24 | 0.9 |  | T1057-D1 | 90.24 | 0.84 |  | T1057-D1 | 90.24 | 0.85 |
| T1058-D1 | 89.22 | 0.82 |  | T1058-D1 | 89.22 | 0.82 |  | T1058-D1 | 89.22 | 0.83 |
| T1058-D2 | 75.31 | 0.71 |  | T1058-D2 | 75.31 | 0.64 |  | T1058-D2 | 75.31 | 0.71 |
| T1060s2-D1 | 91.95 | 0.72 |  | T1060s2-D1 | 91.95 | 0.67 |  | T1060s2-D1 | 91.95 | 0.66 |
| T1060s3-D1 | 98.39 | 0.69 |  | T1060s3-D1 | 98.39 | 0.73 |  | T1060s3-D1 | 98.39 | 0.74 |
| T1061-D1 | 86.21 | 0.52 |  | T1061-D1 | 86.21 | 0.51 |  | T1061-D1 | 86.21 | 0.52 |
| T1061-D2 | 74.26 | 0.51 |  | T1061-D2 | 74.26 | 0.54 |  | T1061-D2 | 74.26 | 0.52 |
| T1061-D3 | 84.62 | 0.68 |  | T1061-D3 | 84.62 | 0.68 |  | T1061-D3 | 84.62 | 0.68 |
| T1064-D1 | 6.52 | 0.25 |  | T1064-D1 | 6.52 | 0.25 |  | T1064-D1 | 6.52 | 0.24 |
| T1065s1-D1 | 71.67 | 0.84 |  | T1065s1-D1 | 71.67 | 0.83 |  | T1065s1-D1 | 71.67 | 0.82 |
| T1065s2-D1 | 89.8 | 0.84 |  | T1065s2-D1 | 89.8 | 0.84 |  | T1065s2-D1 | 89.8 | 0.84 |
| T1067-D1 | 57.66 | 0.54 |  | T1067-D1 | 57.66 | 0.54 |  | T1067-D1 | 57.66 | 0.54 |
| T1068-D1 | 46.67 | 0.65 |  | T1068-D1 | 46.67 | 0.57 |  | T1068-D1 | 46.67 | 0.6 |
| T1070-D1 | 0 | 0.35 |  | T1070-D1 | 0 | 0.36 |  | T1070-D1 | 0 | 0.35 |
| T1070-D2 | 19.61 | 0.87 |  | T1070-D2 | 19.61 | 0.87 |  | T1070-D2 | 19.61 | 0.87 |
| T1070-D3 | 44.74 | 0.61 |  | T1070-D3 | 44.74 | 0.6 |  | T1070-D3 | 44.74 | 0.61 |
| T1070-D4 | 79.41 | 0.72 |  | T1070-D4 | 79.41 | 0.72 |  | T1070-D4 | 79.41 | 0.72 |
| T1073-D1 | 60 | 0.77 |  | T1073-D1 | 60 | 0.77 |  | T1073-D1 | 60 | 0.78 |
| T1074-D1 | 46.97 | 0.52 |  | T1074-D1 | 46.97 | 0.56 |  | T1074-D1 | 46.97 | 0.57 |
| T1076-D1 | 94.81 | 0.92 |  | T1076-D1 | 94.81 | 0.95 |  | T1076-D1 | 94.81 | 0.94 |
| T1078-D1 | 67.69 | 0.83 |  | T1078-D1 | 67.69 | 0.84 |  | T1078-D1 | 67.69 | 0.81 |
| T1079-D1 | 87.17 | 0.83 |  | T1079-D1 | 87.17 | 0.75 |  | T1079-D1 | 87.17 | 0.82 |
| T1080-D1 | 2.99 | 0.45 |  | T1080-D1 | 2.99 | 0.45 |  | T1080-D1 | 2.99 | 0.45 |
| T1082-D1 | 39.47 | 0.49 |  | T1082-D1 | 39.47 | 0.51 |  | T1082-D1 | 39.47 | 0.5 |
| T1083-D1 | 97.83 | 0.82 |  | T1083-D1 | 97.83 | 0.83 |  | T1083-D1 | 97.83 | 0.82 |
| T1084-D1 | 88.89 | 0.86 |  | T1084-D1 | 88.89 | 0.82 |  | T1084-D1 | 88.89 | 0.85 |
| T1087-D1 | 44.68 | 0.42 |  | T1087-D1 | 44.68 | 0.41 |  | T1087-D1 | 44.68 | 0.41 |
| T1089-D1 | 74.6 | 0.78 |  | T1089-D1 | 74.6 | 0.81 |  | T1089-D1 | 74.6 | 0.81 |
| T1090-D1 | 38.95 | 0.57 |  | T1090-D1 | 38.95 | 0.62 |  | T1090-D1 | 38.95 | 0.61 |
| T1091-D1 | 94.29 | 0.73 |  | T1091-D1 | 94.29 | 0.74 |  | T1091-D1 | 94.29 | 0.72 |
| T1091-D2 | 85.19 | 0.71 |  | T1091-D2 | 85.19 | 0.77 |  | T1091-D2 | 85.19 | 0.76 |
| T1091-D3 | 88.68 | 0.76 |  | T1091-D3 | 88.68 | 0.72 |  | T1091-D3 | 88.68 | 0.73 |
| T1091-D4 | 14.29 | 0.82 |  | T1091-D4 | 14.29 | 0.84 |  | T1091-D4 | 14.29 | 0.84 |
| T1092-D1 | 51.22 | 0.58 |  | T1092-D1 | 51.22 | 0.47 |  | T1092-D1 | 51.22 | 0.46 |
| T1092-D2 | 76.92 | 0.68 |  | T1092-D2 | 76.92 | 0.79 |  | T1092-D2 | 76.92 | 0.8 |
| T1093-D1 | 8.45 | 0.4 |  | T1093-D1 | 8.45 | 0.37 |  | T1093-D1 | 8.45 | 0.38 |
| T1093-D2 | 33.51 | 0.5 |  | T1093-D2 | 33.51 | 0.64 |  | T1093-D2 | 33.51 | 0.62 |
| T1093-D3 | 0 | 0.2 |  | T1093-D3 | 0 | 0.16 |  | T1093-D3 | 0 | 0.16 |
| T1094-D1 | 46.04 | 0.47 |  | T1094-D1 | 46.04 | 0.59 |  | T1094-D1 | 46.04 | 0.59 |
| T1094-D2 | 19.23 | 0.28 |  | T1094-D2 | 19.23 | 0.28 |  | T1094-D2 | 19.23 | 0.28 |
| T1095-D1 | 60.8 | 0.51 |  | T1095-D1 | 60.8 | 0.67 |  | T1095-D1 | 60.8 | 0.65 |
| T1096-D1 | 38.28 | 0.69 |  | T1096-D1 | 38.28 | 0.65 |  | T1096-D1 | 38.28 | 0.64 |
| T1096-D2 | 48.84 | 0.74 |  | T1096-D2 | 48.84 | 0.72 |  | T1096-D2 | 48.84 | 0.77 |
| T1099-D1 | 19.1 | 0.51 |  | T1099-D1 | 19.1 | 0.55 |  | T1099-D1 | 19.1 | 0.55 |
| T1100-D1 | 38.82 | 0.67 |  | T1100-D1 | 38.82 | 0.67 |  | T1100-D1 | 38.82 | 0.67 |
| T1100-D2 | 58.23 | 0.64 |  | T1100-D2 | 58.23 | 0.64 |  | T1100-D2 | 58.23 | 0.64 |
| T1101-D1 | 90.48 | 0.84 |  | T1101-D1 | 90.48 | 0.84 |  | T1101-D1 | 90.48 | 0.84 |
| T1101-D2 | 86.92 | 0.74 |  | T1101-D2 | 86.92 | 0.75 |  | T1101-D2 | 86.92 | 0.74 |
